# Target the Host, Kill the Bug; Targeting Host Respiratory Immunosuppressive Responses as a Novel Strategy to Improve Bacterial Clearance During Lung Infection

**DOI:** 10.3389/fimmu.2020.00767

**Published:** 2020-04-30

**Authors:** Alanna M. Kelly, Rachel M. McLoughlin

**Affiliations:** Host-Pathogen Interactions Group, School of Biochemistry and Immunology, Trinity Biomedical Sciences Institute, Trinity College Dublin, Dublin, Ireland

**Keywords:** immunosuppression, lung, chronic infection, host-directed therapies, bacterial infection

## Abstract

The lung is under constant pressure to protect the body from invading bacteria. An effective inflammatory immune response must be tightly orchestrated to ensure complete clearance of any invading bacteria, while simultaneously ensuring that inflammation is kept under strict control to preserve lung viability. Chronic bacterial lung infections are seen as a major threat to human life with the treatment of these infections becoming more arduous as the prevalence of antibiotic resistance becomes increasingly commonplace. In order to survive within the lung bacteria target the host immune system to prevent eradication. Many bacteria directly target inflammatory cells and cytokines to impair inflammatory responses. However, bacteria also have the capacity to take advantage of and strongly promote anti-inflammatory immune responses in the host lung to inhibit local pro-inflammatory responses that are critical to bacterial elimination. Host cells such as T regulatory cells and myeloid-derived suppressor cells are often enhanced in number and activity during chronic pulmonary infection. By increasing suppressive cell populations and cytokines, bacteria promote a permissive environment suitable for their prolonged survival. This review will explore the anti-inflammatory aspects of the lung immune system that are targeted by bacteria and how bacterial-induced immunosuppression could be inhibited through the use of host-directed therapies to improve treatment options for chronic lung infections.

## Introduction

The respiratory tract is in constant contact with a myriad of bacterial species. To maintain homeostasis the healthy lung must efficiently and precisely identify friend from foe, and defend from infection without any long lasting inflammation or immunopathology occurring. The lung employs a complex network of immunosuppressive responses which are critical for maintaining a stable tolerogenic microenvironment within the tissue. However, bacterial pathogens have evolved to establish themselves within the lung by targeting this immunosuppressive arm of the immune response. In doing so bacterial species prevent appropriate clearance and create a suppressive microenvironment, facilitating their long-term survival. A better understanding of these bacterial-induced responses may aid in the development of novel host-directed therapies (HDTs) to target bacterial-induced immunosuppression. Herein we discuss how bacterial species of the lung have evolved to manipulate host immunosuppressive responses to their own advantage, hijacking important tolerogenic mechanisms to create an environment appropriate for bacterial growth. In addition we will discuss the ongoing research into HDTs and their potential for improving current treatments against bacterial lung infections.

Bacterial-induced respiratory tract infections are a major global health concern, and represent leading causes of death worldwide. These infections are of particular concern due to our dense population living habits and the growing prevalence of community-acquired pneumonia (1). The discovery and mass production of antibiotics dramatically reduced the number of mortalities associated with bacterial infection. However, with growing resistance and a lack of new discoveries, multi-drug resistance (MDR) is a huge threat to the success of antibiotics. The world health organization (WHO) has drawn up an antibiotic-resistant “priority pathogen” list classifying MDR bacteria as “critical” and “priority” depending on the threat to human health and the need for the development of novel antibiotics against these bacteria. Many of those in the “critical” group are bacteria associated with infection of the lung such as *Mycobacterium tuberculosis, Pseudomonas aeruginosa* and *Klebsiella pneumoniae*, with other species on the “priority” list including *Staphylococcus aureus, Streptococcus pneumonia* and *Haemophilus influenza*, all important bacterial species associated with invasive lung infections (2).

Our dependence on antibiotics is not sustainable and alternative therapies to current antibiotic treatments are urgently required to combat these increasingly dangerous pathogens and to prevent chronic and often fatal lung infections. As the resistance problem grows many researchers have looked into the modification of existing antibiotics to improve activity and reduce sensitivity to resistance (3). This approach has been broadly successful, however, current methods of targeting the microbe using antibiotics seem to inevitably end in resistance to the therapy in use. A potential method to overcome this may be the development of novel HDTs whereby the local host immune response during infection is targeted. Targeting host immunity can be done to either improve the immune response to infection or dampen down/disarm inappropriate immune responses, this includes strategies employed by bacteria to exploit immunosuppressive mechanisms of the host. By dampening local bacterial-induced immunosuppression it may be possible to promote a more pro-inflammatory immune response leading to improvements in bacterial killing and clearance. Achieving this however requires an in depth understanding of the intricacies of how the bacteria interact with the host to subvert immunosuppressive responses. This may reveal potential targets to intercept for the development of novel treatment modalities against intractable respiratory infections.

## Immune Response in the Lung During Bacterial Infection

Pulmonary immune homeostasis is critical in maintaining a healthy lung environment and is tightly regulated by a network of tissue-resident and infiltrating immune cells that monitor the external environment continuously (4). The lung immune system has evolved to ward off pathogenic invasion while simultaneously preventing inflammation-mediated damage. An effective pro-inflammatory immune response is vital for the successful and complete elimination of bacterial pathogens. This involves a complex network of resident and infiltrating innate and adaptive inflammatory immune cells in addition to the epithelial cells lining the conducting airways and alveolar surface, which come together to orchestrate the efficient clearance of invading bacteria from the lung. These essential inflammatory immune cell responses are summarized in Table 1.

**Table 1:** Important inflammatory immune responses of the lung vital for bacterial clearance.

**Cell type**	**Function**	**References**
Alveolar macrophage (AM)	1st line defense. Phagocytosis of infiltrating pathogens Production of inflammatory cytokines; IL-12, TNF, IL-1β, IL-8	(5–7)
Dendritic cell (DC)	Phagocytosis of pathogens & upregulation of MHC-II, presentation of bacterial antigens, and directing T effector cell differentiation. Production of inflammatory cytokines; IL-12, TNF, IL-1β	(6, 8, 9)
Neutrophil	Facilitate bacterial clearance via phagocytosis, enzymatic degradation of bacteria and NETosis	(7, 10, 11)
Natural Killer (NK) cell	Early contribution to high IFN-γ levels and promotion of Th1 responses, promote inflammatory macrophage responses	(12–14)
Innate lymphoid (ILC) cell	Contribute to the production of IL-17 and IFN-γ in the lung, leading to enhanced pro-inflammatory innate responses. Produce IL-22 promoting antimicrobial peptide production	(15–17)
Natural Killer T (NKT) cell	Contribute to high IFN-γ levels and promotion of Th1 responses, IL-17 production leading to neutrophil recruitment	(18–21)
Mucosal-associated invariant T (MAIT) cell	Contribute to IFN-γ and promotion of Th1 responses, IL-17 production resulting in neutrophil recruitment and aid in recruitment of CD4^+^ and CD8^+^ T cells	(22–24)
γδ T cell	Major producers of IL-17 leading to neutrophil-mediated responses. Contribute to IFN-γ production	(25, 26)
CD4^+^ T cell	Critical contributors to the production of IL-17, IL-22 and IFN-γ, leading to enhanced innate cell activity, antimicrobial peptide production, and improved bacterial clearance	(27–31)
CD8^+^ T cell	Contribute to IFN-γ and TNF production. Cytotoxic effects help clear infected cells and remove bacteria from the lung	(32–34)

Once the bacterial pathogen is cleared from the respiratory tract it is vital the inflammatory immune response is quickly subdued to prevent any further tissue damage or immunopathology from occurring. The resolution of the inflammatory pulmonary immune response is an integral part of the physiological response to tissue injury and infection in the lungs. Inappropriate acute or long-term chronic inflammatory responses lead to damage of the thin-walled organ and result in impaired gas exchange and possibly life-threatening lung failure (35, 36). The termination of inflammatory immune responses is tightly regulated by a network of immunosuppressive cells including: alternatively activated alveolar macrophages (AM), myeloid-derived suppressor cells (MDSCs), tolerogenic dendritic cell (DC) subsets, IL-10-producing CD4^+^ T cells, regulatory T cells (Tregs), regulatory B cells (Bregs), and lung epithelial cells (37–42), and through the release of key immunosuppressive cytokines. Immunoregulatory cells and cytokines act to prevent excessive inflammation by abrogating signaling pathways needed for inflammatory cytokine production, depleting populations of inflammatory effector cells and altering the phenotypes of leukocytes to an anti-inflammatory state (4, 43–47). In doing so, there is a return to homeostasis after a bacterial infection of the lung with minimal damage to the tissue.

## Manipulation of the Anti-Inflammatory Immune Responses Of The Host Lung

The resolution of inflammation is vital to ensure lung health, however many pulmonary bacterial pathogens have evolved to undermine these regulatory immune responses and use them against the host for their own survival.

### Manipulation of Immunoregulatory Cells

#### Alveolar Macrophages

The plasticity of the alveolar macrophage (AM) response is critical to their role in the clearance of bacteria from the lung and the resolution of inflammation post-infection, but also makes these cells ideal targets for hijacking by many bacteria. While the prevalence of the “tissue repair” M2 phenotype is needed for resolution of inflammation, these cells represent a niche for the prolonged survival of many intracellular pathogens (48). In murine studies *M. tuberculosis* increases the expression of peroxisome proliferator-activated receptor-γ (PPAR-γ) in infected macrophages leading to an increase in anti-inflammatory “M2”-associated markers alongside reductions in respiratory burst, allowing enhanced intracellular bacterial survival (49). *Mycobacterium tuberculosis* has also been shown to induce arginase1 (Arg1) expression in infected macrophages which is associated with reduced production of reactive nitrogen intermediates and therefore enhanced survival of the bacterium (50). AMs are also polarized to an M2 phenotype during *Bordetella pertussis* intracellular infection to facilitate survival of the bacteria within these cells (51). *In vitro* studies using a THP-1 cell line demonstrated that *B. pertussis* can persist in macrophages and promote the expression of suppressor of cytokine signaling 1(SOCS1) protein, an M2-associated protein (52). The upregulation of SOCS1 promotes Arginase-1 (Arg1) activity and inhibits IFN-γ induced JAK2/STAT1 signaling and TLR/NF-kB signaling leading to reduced pro-inflammatory responses (53, 54). Similarly the bacterial toxins Pertussis toxin (Ptx) and adenylate cyclase toxin (ACT) were implicated in this macrophage phenotype switch. *In vitro* studies have demonstrated that THP-1 cells infected with strains lacking either of these toxins had lower SOCS1 expression and a decreased ability of the bacterium to survive intracellularly (51).

#### Dendritic Cells

Dendritic cells (DCs) have a decisive role in initiating an appropriate adaptive immune response to invading pathogens in the lung (55), while also being central to tolerogenic responses and inflammatory resolution. The induction of tolerogenic DCs is an effective method of manipulating the lung immune response employed by a number of bacterial species in order to allow the pathogen to multiply without restraint. *Yersinia pestis* promotes the expansion of tolerogenic DCs via its LcrV protein (56). *In vitro* studies using bone marrow-derived DCs (BMDCs) have shown LcrV binds TLR2/6 leading to the induction of high levels of IL-10 production by these cells which in turn promotes type 1 regulatory (Tr1) T cells and further enhanced IL-10 production (56). Similarly the induction of tolerogenic DCs were also seen during Mycobacterium *avium* subspecies *hominissuis* (MAH) co-infection (57). MAH infections are strongly associated with opportunistic co-infections by common pulmonary pathogens such as *Haemophilus influenzae, Staphylococcus aureus*, and *Pseudomonas aeruginosa* (57, 58). Studies using MAH-infected BMDCs stimulated with LPS, which mimicked co-infection conditions, lead to the production of high levels of TLR-mediated IL-10 alongside reduced IL-12 levels (57). *In vitro* studies of a MAH/*P. aeruginosa* co-infection showed a marked increase in IL-10-producing tolerogenic DCs. The enhanced IL-10 led to reduced MHC class II expression and antigen presentation, which eventually led to the inhibition of CD4^+^ T cell proliferation (57). By promoting tolerogenic phenotypes of AMs and DCs in the lung bacteria can promote early IL-10 production and reduced antigen-presentation resulting in the prevention of effective protective pro-inflammatory adaptive responses leading to undisturbed bacterial growth.

#### Myeloid-Derived Suppressor Cells

Myeloid-derived suppressor cells (MDSCs) are emerging as key specialized suppressive cells capable of dampening inflammation to prevent tissue damage after infection (59). These cells are powerful modulators of both the innate and adaptive immune responses and in particular have potent immunosuppressive effects on T cell responses (60). These immunosuppressive innate cells have been targeted by a number of pulmonary bacteria which lead to the progression of chronic infections and these cells may be particularly important in facilitating the transition from acute to chronic infection (61–63).

MDSC are increased in the peripheral blood of patients with active tuberculosis infection (63). *In vitro* studies using a granuloma model demonstrate how MDSCs exposed to *M. tuberculosis* secrete IL-10 in abundance and upregulate their expression of PD-L1, which led to the suppression of protective T cell proliferation and promoted bacterial replication (64). The bacterium *Streptococcus pneumoniae* also has the capacity to hijack MDSCs to facilitate its persistence in the airways. Studies in mice have demonstrated a robust monocytic response in the lung following intranasal challenge with *S. pneumoniae* which was dominated by the presence of MDSCs. These cells expressed IL-10, arginase and importantly lacked phagocytic capabilities (65). This early anti-inflammatory response terminated pro-inflammatory signaling needed for clearance of the bacteria and promoted persistence in the lung. Similarly the expansion of a large population of regulatory immature myeloid cells has been described following intranasal *Francisella tularensis* infection (66). A lethal *F. tularensis* infection with a highly virulent strain, led to the recruitment of a large number of MDSC to the lungs which allowed bacterial survival and eventually resulted in host death. Interestingly, during sub-lethal infection of *F. tularensis* there was a greater recruitment of mature pro-inflammatory myeloid cells that were effective at controlling infection and clearing the bacterium (66). The depletion of the immature myeloid cells partially ameliorated mortality following lethal infection. These results indicate the lethality of *F. tularensis* infection may be through excessive MDSC recruitment which enables prolific bacterial growth within the lungs. Other bacterial species which colonize and infect the respiratory tract may also have the potential to similarly subvert the activities of MDSCs. In a systemic infection model of *S. aureus* infection, persistence of the bacterium has been shown to be associated with increased expansion of MDSCs which inhibit T cell responses (67). Similarly in a localized skin infection model *S. aureus* promotes the expansion of MDSCs leading to the upregulation of IL-10 production, which was associated with the persistence of the bacteria within the skin (68).

#### Treg Cells

Treg cells play a particularly important role in maintaining lung homeostasis and resolving pro-inflammatory responses promptly after pathogens have been cleared. Many pathogens that invade the lungs have the capacity to exploit the immunoregulatory function of Tregs and promote the expansion of these anti-inflammatory cells. This strategy is a direct attempt to counterbalance the pro-inflammatory effects mediated by innate cells and adaptive T cells such as Th17 cells and Th1 cells which play a particularly important role in the protective immune response to bacteria in the lung (27, 69–71). Tregs can carry out their function through the production of immunoregulatory cytokines such as IL-10 and TGF-β, via direct cell-cell contact through their immunosuppressive surface markers such as CTLA-4 or by their enhanced consumption of IL-2 which reduces effector T cell activation (72).

Peripheral blood mononuclear cells (PBMCs) from patients with active tuberculosis were shown to have raised levels of Tregs in comparison to healthy controls (73).The *ex vivo* depletion of these CD25^+^ FoxP3^+^ cells from PBMCs of patients led to an increased expansion of antigen-specific IFN-γ^+^ T cells indicating that *M. tuberculosis*–induced Tregs were capable of suppressing these protective T cell responses. In a guinea pig model of tuberculosis it was shown that highly virulent strains induce high levels of FoxP3, IL-10, and TGF-β mRNA expression in lung tissue (74, 75). After an initial increase in mRNA expression levels of the Th1-associated cytokine IFN-γ, there was a rapid decrease in these levels and subsequent surge in Treg-associated markers and cytokines (74). It was hypothesized that highly virulent strains act as potent inducers of Tregs and that this increase in these suppressive cells enhances bacterial survival. A similar result was seen in a murine model of severe tuberculosis infection using a hyper-virulent strain HN878. An initial Th1 response was followed by a rapid increase in IL-10^+^ Tregs in the lungs (76). This increase in suppressive cells enabled progression of infection which may explain the relatively short time of survival of these infected mice.

Other bacterial species such as *B. pertussis* and *S. pneumoniae* also promote Treg expansion as a means to facilitate their survival. *B. pertussis* can expand Tregs by upregulating IL-10 production by innate cells such as macrophages and DCs, which in turn directs T cell differentiation toward a regulatory phenotype (77–80). In a murine model of *S. pneumoniae* nasopharyngeal colonization it was demonstrated that Treg expansion was due to the high levels of TGF-β being produced by nasopharyngeal cells in response to the bacterium. The high levels of Treg cells promote prolonged survival of the bacteria within the host lungs while causing no damage to the host (81). The bacterium essentially goes undetected by the pro-inflammatory arm of the pulmonary immune response as it remains suppressed by the presence of high levels of TGF-β. This lack of inflammatory responses via Treg-mediated TGF-β allows bacterial survival and may lead to invasive disease.

#### Breg Cells

In addition to the well-characterized role of Tregs in maintaining immune homeostasis, Bregs have been shown to contribute to immune tolerance (82, 83). Their suppressive effect is mainly through IL-10 production, leading to the inhibition of Th1 and Th17 responses and the conversion of CD4^+^ T cells into Treg cells (82, 84). The role of Bregs during pulmonary infection is not well defined, however PBMCs from patients suffering with tuberculosis were shown to contain higher numbers of Bregs compared to healthy controls (85). *In vitro* co-cultures using T cell and B cell populations isolated from patient PBMCs demonstrated that these Bregs could dampen Th17 responses (85). Bregs were also shown to have inhibitory effects on IL-22 production (86), a cytokine implicated in limiting *M. tuberculosis* intracellular survival (87, 88). More recent studies have demonstrated how circulating Bregs of active *M. tuberculosis*-infected patients were producing the immunoregulatory cytokine IL-35 (89), a potent immunosuppressive cytokine capable of suppressing effector T cell responses, promoting the expansion of Tregs and their production of IL-10 (90). Additionally, stimulating purified B cells from healthy controls with *M. tuberculosis* lysate, increased expansion of IL-35^+^ Bregs (89), suggesting *M. tuberculosis* may be inducing the production of IL-35 by Bregs to enhance IL-10 production to regulate inflammatory responses. More in depth study is needed to definitively identify the function of IL-35-producing B cells in *M. tuberculosis* infection. In a murine model of *S. aureus* systemic infection a population of Bregs were expanded and acted as major producers of IL-10 (68). It is conceivable therefore that these cells also play a role in the pathology of *S. aureus* lung infection.

There are still major gaps in our understanding of how Bregs and their immunosuppressive effects are being hijacked by pulmonary bacteria, however, it seems likely that these cells are important in suppressing inflammatory immune responses in the lung through their production of the anti-inflammatory cytokines IL-10 and potentially IL-35.

Regulatory cell populations of the local respiratory immune response are key targets of bacterial pathogens. Immune cells usually central to the resolution of harmful inflammation are heavily exploited to improve bacterial survival. Bacterial-induced expansion and activation of these regulatory innate and adaptive immune cells allows the suppression of vital inflammatory responses needed for complete bacterial clearance. These cells could potentially be targeted to reduce their anti-inflammatory effects and improve infection outcome.

### Manipulation of Immunosuppressive Cytokines

One of the most common survival strategies employed by bacterial species to facilitate their survival is to promote the production of various anti-inflammatory cytokines from a variety of cell types. These anti-inflammatory mediators then suppress pro-inflammatory cell populations such as innate cells and effector T cells, and reduce the production of pro-inflammatory cytokines.

#### IL-10

IL-10 is a key cytokine required for maintaining the steady-state within the healthy lung during infection where it inhibits the activity of many pro-inflammatory cells and prevents immunopathology. However, excessive or miss-timed IL-10 production can inhibit a protective pro-inflammatory response leading to chronic and often fatal infection (91). In a murine model of *Mycobacterium avium* infection, BALB/c mice respond with very early IL-10 production. This reduces their ability to control the pathogen compared to their C57BL/6 counterparts, which have lower IL-10 activity (92). The ablation of IL-10 signaling improved pathogen control in the BALB/c mice, highlighting the causal relationship between IL-10 and a lack of pathogen control. By limiting pro-inflammatory cell functions IL-10 can reduce immunopathology, benefitting the host. However, if produced in excess or if inappropriately timed, it can potentially dampen the protective effector immune response required for bacterial clearance.

The induction of IL-10 is a key strategy employed by *M. tuberculosis* to facilitate pathogenesis (93). The pathogen infiltrates the lung and can reside within host macrophages, here, it suppresses their pro-inflammatory function through enhanced IL-10 production via a number of mechanisms (94). The *M. tuberculosis* heat shock protein 60 (Mtbhsp60) can target TLR2 and TLR4 on macrophages leading to p30 MAPK activation and enhanced IL-10 production (95). By targeting TLR signaling *M. tuberculosis* can polarize macrophage responses to be more immunosuppressive. Das et al. identified a novel mechanism of CCR5–mediated altered cellular signaling in *M. tuberculosis* infected macrophages, leading to downstream activation of the Src kinase Lyn and the mitogen-activated protein (MAP) kinase ERK-1/2, resulting in IL-10, and also TGF-β production. The elevated IL-10 production was shown to attenuate the expression of MHC-II in the infected macrophages (94), reducing the ability of these cells to orchestrate an appropriate adaptive response. Enhanced IL-10 production from *M. tuberculosis*-infected macrophages has also been shown to inhibit phagosomal maturation which enables phagosomal escape and intracellular survival within the host lung, further contributing to impaired bacterial clearance (96–98). Furthermore, this arrest of maturation prevents appropriate presentation of antigenic peptides and subsequent induction of the adaptive immune response (99). Blocking IL-10 production *in vitro* in AMs, by pre-treating them with an anti-IL-10 antibody in advance of *M. tuberculosis* infection, was shown to improve phagosome maturation and increase killing of the internalized mycobacterium (98). Alveolar epithelial cells are also targeted by *M. tuberculosis* for IL-10 production. *Mycobacterium bovis* Bacillus Calmette Guerin (BCG) activates TLR2 and TLR4 on alveolar epithelial cells causing phosphorylation of glycogen synthase kinase-3 (GSK3) via a PI3K/Akt pathway which induces the production of IL-10 (100). *M. tuberculosis* can also manipulate IL-10 regulation at the RNA level. *M. tuberculosis* drives the anti-inflammatory microRNA (miRNA) miR-21 (101), which is one of the most highly expressed miRNAs in myeloid cells. This miRNA is induced by TLR4 signaling and limits the activity of the pro-inflammatory PDCD4 protein to promote IL-10 production (102). *M. tuberculosis* also has the capacity to activate IL-10-expressing T cells (103). T cells are the predominant producers of IL-10 later in *M. tuberculosis* infection, at ~21 days post-infection, where they contribute to impaired clearance (103).

Other bacterial species, such as *B. pertussis*, utilize a number of virulence factors to drive IL-10 production (78, 79, 104, 105). Filamentous haemaglutinin (FHA) activates TLR4 signaling in DCs and macrophages within the lung to induce IL-10 (78, 104). This increase in innate IL-10 leads to the expansion of T regulatory type 1 (Tr1) cells (78). FHA-induced IL-10 also dampens IL-12 production by DCs and leads to downregulation of protective Th17 and Th1 responses (79). However, it must be noted that more recent research has cast a question mark over the immunoregulatory role of FHA (106). The authors state that the upregulation of IL-10 may be caused by endotoxin contamination or co-purification of bacterial lipoproteins, which may contribute to FHA's cytokine inducing activity (106, 107). Adenylate cyclase toxin (ACT) of *B. pertussis* also drives IL-10 production by APCs such as DCs and macrophages (77, 108), which then further induces IL-10-secreting Treg cells (109).

Klebsiella *pneumoniae* can induce IL-10 production by macrophages in the lung (110), which enables the bacterium to inhibit inflammasome activation and pyroptotis, facilitating its' dissemination (111). Upon infection with *K. pneumoniae* strain A54970, no IL-1β production was induced in infected macrophages, suggesting a lack of inflammasome activation and associated pyroptosis (111, 112). When BMDMs from IL-10 knock-out (KO) mice were infected with the A54970 strain, these macrophages produced high levels of IL-1β and released LDH which is associated with the induction of pyroptosis, indicating that this strain was capable of inhibiting inflammasome activation and function through high induction of IL-10 (111, 113). However, the authors noted that different clinical strains investigated showed dissimilar methods of evading the host immune response. Other strains such as A28006 induced high IL-1β production and increased pyroptotic cell death in macrophages recruited to the lung (111). In a murine model of *K. pneumoniae* infection, intra-tracheal challenge with *K. pneumoniae* enhanced IL-10 mRNA expression in lung homogenates. Administration of IL-10-blocking antibodies prior to challenge was associated with an enhanced pro-inflammatory immune response, improved bacterial clearance and prolonged survival (114). It has been postulated that the capsular polysaccharide (CPS) of *K. pneumoniae* may be particularly important for the induction of IL-10 (110, 115). When mice were intranasally inoculated with a capsulated or a non-capsulated strain of *K. pneumoniae*. IL-10 levels in the bronchiolar lavage fluid (BALF) and serum of mice infected with the capsulated strain were significantly higher than those infected with the non-capsulated strain, and these mice died within 3 days of infection (110). These results suggest that the presence of CPS is inducing IL-10 production at the site of infection and may down-regulate the expression of pro-inflammatory cytokines such as TNF and IFN-γ. The multiple cellular sources of IL-10 *in vivo* and the specific signaling pathways controlling *Klebsiella*-induced IL-10 production are areas of research warranting future investigation (116).

Bacteria of the lung can also promote TLR-driven IL-10 production to facilitate their survival. Species of *Yersinia* and *S. aureus* drive IL-10 production through the exploitation of TLR2 signaling. Studies have demonstrated that *Yersinia* species upregulate host macrophage-derived IL-10 production in a TLR-2-mediated manner, resulting in suppression of pro-inflammatory cytokine production by macrophages and increased bacterial survival (117, 118). Virulence factor LcrV-induced IL-10 was shown to induce hypo-responsiveness against TLR2- or TLR4-agonists in macrophages. This hypo-responsiveness was not present in cells isolated from IL-10 KO mice (119). This result demonstrates how the bacterium is exploiting an IL-10-induced TLR tolerance mechanism of the host. Likewise, the Gram-positive bacterium *S. aureus* can drive IL-10 via TLR signaling. In addition to conventional pro-inflammatory signaling induced through TLR2 recognition of *S. aureus*, the bacteria is also capable of inducing a robust anti-inflammatory response (120). *Staphylococcus aureus*-induced TLR2 signaling in monocytes has been shown to result in PI3K/Akt signaling leading to IL-10 production, as opposed to conventional NFκB-driven TLR2 signaling which leads to pro-inflammatory cytokine production (121). The anti-inflammatory signaling response is induced independently of the pro-inflammatory response, and can be “uncoupled” from these inflammatory responses (120). It has previously been shown that *S. aureus* can induce IL-10 production in a skin infection model to promote its persistence by inhibiting effector T cells (68), suggesting that a similar mechanism may occur during *S. aureus* infection and/or colonization of the respiratory tract. *S. aureus* is a common cause of secondary pneumonia following influenza infection and Robinson et al. demonstrated a potential role for both IL-27 and IL-10 in impairing bacterial clearance in a murine model of secondary *S. aureus*-induced pneumonia (122). IL-10 KO mice cleared the bacteria from the lung more efficiently than WT mice. IL-27RA KO mice had decreased levels of IL-10 which was associated with improved bacterial clearance compared to WT counterparts (122). These results indicate that IL-10 is facilitating *S. aureus* persistence in the lung post influenza infection, and that IL-27 may have a role to play in regulating the production of IL-10 in this instance.

#### TGF-β

Although the induction of IL-10 represents one of the most heavily exploited immunosuppressive strategies used by bacteria to facilitate their persistence in the lung, other regulatory cytokines have also been implicated in bacterial-induced immunosuppression. Upregulation of TGF-β is commonly associated with *S. pneumoniae* carriage and lung infection. Adenoidal mononuclear cells isolated from children undergoing adenoidectomies who also tested positive for *S. pneumoniae* carriage had higher levels of TGF-β and upregulated populations of FoxP3^+^ Tregs compared to children who tested negative for the bacterium (123). It is thought that these increased immunosuppressive responses within the adenoid tissue may be facilitating chronic carriage, which is a risk factor for pneumococcal disease. It was demonstrated that the virulence factor enzyme neuraminidase A (NanA) of *S. pneumoniae* can directly activate latent TGF-β to its biologically active form by removal of sialic acids from the latency associated peptide (LAP) (124). TGF-β is associated with prolonged colonization of the nasopharynx and enhanced translocation of *S. pneumoniae* to the lower respiratory tract (125). Furthermore, *S. pneumoniae* infection of Vβ6 integrin KO mice resulted in enhanced clearance of the bacterium compared to WT mice. This was also true in the case of co-infections of influenza and *S. pneumoniae* (126). The αVβ6 integrin is expressed at low levels in healthy tissue but is upregulated in response to inflammation and injury and is the main method of activation of endogenous TGF-β in the lung (127). The complete ablation of β6 integrin from the system led to a loss of activated TGF-β which was accompanied by increased activation of AMs and type I IFN production, resulting in improved protection (126). However, other studies have shown that reduction of TGF-β during *S. pneumoniae* infection can be fatal and that the cytokine is vital in the prevention of hyper-inflammation against *S. pneumoniae* in the lung. Here, blockade of TGF-β led to the dissemination of the bacterium from the lung (128). A caveat of the β6 integrin KO model is that the β6 integrin may also regulate factors other than TGF-β1 that cause the downstream effects facilitating bacterial clearance. TGF-β is clearly critical for limiting infection-associated inflammation, but the powerful immunosuppressive effects TGF-β exerts can be exploited by pulmonary pathogens, with the timing and extent of TGF-β inhibition impacting infection outcome.

Active tuberculosis infection is also associated with the excessive production and activation of TGF-β. TGF-β has been found at high levels in the granuloma of infected patients (129). *In vitro* stimulation of human blood monocytes, with *M. tuberculosis* led to an increase in production of bioactive TGF-β (130). These cells are a likely early source of the cytokine, recruited to the lung upon infection. In a murine model of *M. bovis* pulmonary infection the inhibition of TGF-β by the administration of latency associated peptide (LAP) inhibited TGF-β activity in the lung, enhanced IFN-γ production and improved antigen-specific effector T cell responses in cells isolated from mediastinal lymph nodes of mice administered with LAP compared to PBS-treated mice. LAP treatment was also shown to reduce mycobacterium growth in the lung parenchyma and bronchiolar spaces (131).

#### IL-27

More recently the cytokine IL-27 has been implicated in regulation of the pulmonary immune response during infection. Insights into the function of IL-27 in the lung have primarily come from studies of viral respiratory infections where it appears to play an important role in controlling excessive inflammation. In a murine influenza model, IL-27 suppressed IL-17 production in an IL-10-dependent manner suggesting that IL-27 acts upstream of IL-10 (132). Furthermore, IL-27-mediated activation of STAT1, STAT3, or BLIMP-1 promotes IL-10 production and the generation of T regulatory type 1 (Tr1) cells, leading to the suppression of IL-17 production by CD4^+^ T cells (133–136). Others have demonstrated a direct role for IL-27 in promoting maturation of Tregs during Respiratory syncytial virus (RSV) infection, which was independent of IL-10 (137). In the case of respiratory bacterial infections, IL-27 was identified as an important immune factor underlying the impaired clearance of the bacterium *P. aeruginosa*. In a murine model of *P. aeruginosa* infection this cytokine was shown to suppress the antibacterial activities of AMs (41, 138). Studies demonstrated that a secondary infection with *P. aeruginosa* in the lungs following caecal ligation and puncture (CLP)-induced sepsis in IL-27R KO mice, was associated with enhanced bacterial clearance from the lungs and improved survival rates compared to wild-type counterparts. A more rapid clearance of bacteria from the lungs of the IL-27R KO mice was associated with increased recruitment of inflammatory neutrophils to the airways (138). IL-27 seems to be directly influencing AM responses, as macrophages isolated from IL27R KO mice had enhanced co-stimulatory molecule expression and better bacterial uptake and killing compared to macrophages from wild-type mice (138). Neutralization of IL-27 was also shown to improve bacterial clearance in the lungs of septic mice infected with *P. aeruginosa* (138). Patients suffering from pulmonary infections caused by *Burkholderia pseudomallei* show elevated levels of IL-27 and *in vitro* studies demonstrated that stimulation of whole blood from healthy individuals with *B. pseudomallei* resulted in significantly increased production of IL-27 (139). The major sources of IL-27 were macrophages and neutrophils. The production of IL-27 led to enhanced bacterial survival in neutrophils which was reduced by blockade of neutrophil-derived IL-27 prior to infection (139). Taken together these results suggest that IL-27 has the potential to play an important immunosuppressive role in the lung during bacterial infection and that further studies will reveal the potential for multiple bacterial species to exploit its suppressive effects for their own advantage. One caveat to these studies is, however, the fact that mice have been shown to be capable of producing IL-27p28 in the absence of EBI3. As the subunit may be expressed on its own it is not yet clear if the reported effects of IL-27 are due to the full heterodimeric cytokine or merely to its alpha subunit. However, it has recently been reported that a transgenic mouse that can only produce IL-27p28 in the presence of EBI3 has been developed (140), which will allow for the distinct functions of IL-27 or IL-27p28 to be elucidated.

**Figure 1:**
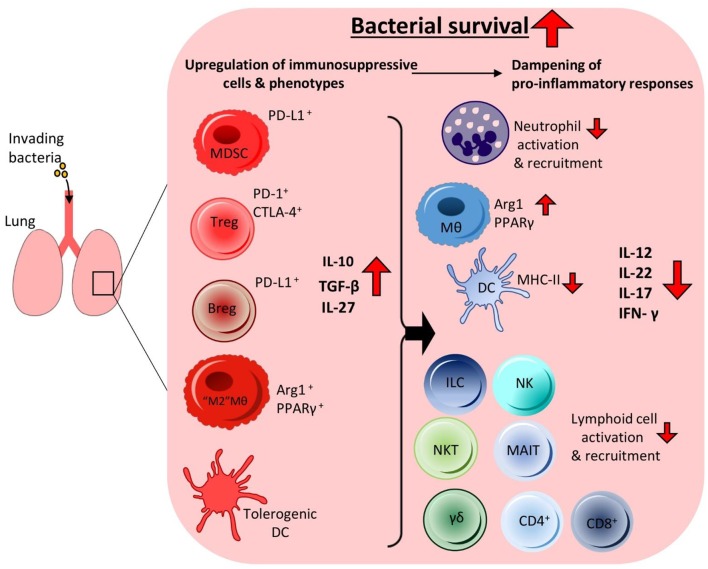
Invading bacteria induce an immunosuppressive microenvironment in the lung resulting in bacterial persistence. To facilitate persistence during infection invading bacteria can promote immunosuppressive immune responses in the lung by targeting the anti-inflammatory arm of the host immune system. This leads to the increased production of anti-inflammatory cytokines and enhanced recruitment of anti-inflammatory cells, which together reduce pro-inflammatory cytokine production and cell populations in the airways. The bacteria can manipulate alveolar macrophages and DCs to produce high levels of regulatory cytokines such as IL-10 and IL-27. This leads to reduced recruitment and activation of inflammatory innate cells such as neutrophils, NK cells, γδ T cells, NKT cells, and MAIT cells. Impaired DC MHC-II expression and higher IL-10 production leads to reduced activation of adaptive effector T cell responses such as Th1, Th17 and cytotoxic CD8^+^ T cells. Enhanced IL-10 production also leads to increases in Treg cell activation and recruitment. Other anti-inflammatory cell populations also undergo enhanced recruitment to the lung, such as Bregs and MDSCs. Together these anti-inflammatory cells contribute to the creation of an immunosuppressive microenvironment that is permissive to bacterial growth enabling prolonged bacterial survival within the lung without effective clearance.

It is clear that many bacterial pathogens of the lung are profiteering from the induction of immunosuppressive cytokines. By taking advantage of their anti-inflammatory effects on the surrounding inflammatory immune response, many bacteria can survive undisturbed. There is an inextricable link between the immunoregulatory cell populations and cytokines being induced by these pathogens. By promoting the expansion of immunoregulatory cells and enhanced production of immunoregulatory cytokines bacteria can undermine the host's local inflammatory immune responses to survive within the lung (Figure 1).

### Manipulation of Immunometabolism

Altering cell phenotype from pro- to anti-inflammatory is one of the key strategies employed by bacteria invading the lung. Metabolic re-programming is crucial for controlling the switching of cell phenotypes and their pro-inflammatory or anti-inflammatory functions (141). During infection metabolic reprogramming has implications for the production of pro-inflammatory and anti-inflammatory immune cell phenotypes and cytokines which control the ability of the host to clear the pathogen. There is emerging evidence that multiple bacteria have the potential to promote immunosuppressive cell phenotypes and cytokine production by targeting the metabolic pathways of these immune cells.

#### Glycolytic Reprogramming

An increase in glycolysis can be seen as a hallmark of metabolic change in immune cells undergoing activation and is associated with pro-inflammatory responses, with macrophages and DCs increasing their glycolytic metabolism when stimulated with LPS (142, 143). In DCs, this metabolic shift from oxidative phosphorylation to glycolysis upon TLR-mediated activation is antagonized by the metabolic regulator AMP kinase (AMPK), and IL-10 production was also shown to prevent this switch by partially preventing TLR-mediated AMPK hypophosphorylation (142). Bacterial-induced IL-10 production during chronic lung infection therefore has the potential to antagonize the TLR-mediated shift to glycolysis in DCs and thus reduce pro-inflammatory adaptive responses and promote bacterial survival.

LPS-induced glucose uptake and glycolysis in macrophages is also impeded by IL-10. IL-10 inhibits glycolysis by suppressing mechanistic target of rapamycin (mTOR) activity through the induction of DDIT4, an mTOR inhibitor (113). Bone marrow-derived macrophages (BMDMs) from IL-10 KO mice stimulated with LPS were shown to have prolonged activation of mTOR complex 1 (mTORC1), of which mTOR is the catalytic subunit, while mTORC1 was quickly suppressed in macrophages of wild-type mice. The addition of exogenous IL-10 restored mTORC1 regulation in IL-10 KO macrophages (113). DDIT4 was shown to be strongly upregulated by IL-10 during LPS stimulation, indicating it was the likely candidate causing IL-10-induced mTOR suppression (113). BMDMs from Ddit4^−/−^ KO mice also showed prolonged mTORC1 activity upon LPS stimulation, similar to IL-10 KO macrophages, however, treatment with exogenous IL-10 failed to inhibit mTORC1 activation suggesting that the inhibition of mTOR signaling by IL-10 is DDIT4-dependent (113). This study revealed a key role of IL-10 in controlling cellular metabolism in macrophages via inhibition of mTORC1 and suggest that high concentrations of IL-10 induced during chronic pulmonary infection could impede macrophage metabolic switching from oxidative phosphorylation to glycolysis. During active *M. tuberculosis* infection the mTOR signaling pathway is suppressed which promotes the proliferation and activation of FoxP3^+^ Tregs cells. PBMCs isolated from patients with active tuberculosis infection showed decreased numbers of CD3^+^ mTOR^+^ cells and increased expression of FoxP3 compared to healthy controls. The enhanced number of Tregs in infected patients was associated with high levels of IL-10 and TGF-β (144).

Glycolytic reprogramming of macrophages is an important step in the early pulmonary defense against *M. tuberculosis* infection (145). Infection of macrophages induces a shift from oxidative phosphorylation to glycolysis which promotes phagocytosis and enhances production of protective pro-inflammatory cytokines such as IL-1β (145). However, *M. tuberculosis* induced production of IL-10 has been linked to the negative regulation of glycolysis in macrophages (113). PPAR-γ is highly upregulated in human and mouse macrophages upon mycobacterial infection (49, 146). PPAR-γ acts to regulate glucose metabolism and fatty acid storage. Increases in PPAR-γ expression leads to enhanced lipid droplet formation within macrophages, used for intracellular growth by the mycobacterium (147), increased expression of IL-10 and the downregulation of pro-inflammatory immune responses against the mycobacterium (148). *M. tuberculosis* can rewire macrophage energy metabolism to support bacterial survival by limiting ATP availability and decelerating flux through glycolysis and the tricarboxylic (TCA) cycle, increasing mitochondrial dependency on fatty acids. The deceleration of glycolysis may be via citrate, a derivative of the TCA cycle which has been seen to accumulate in monocyte-derived macrophages infected with *M. tuberculosis* (147). At high concentrations citrate can inhibit phosphofructokinase which acts as a key enzyme in glycolysis (149). One of the caveats to this work is that different metabolic outcomes occurred depending on whether BCG, heat-killed *M. tuberculosis* or live virulent *M. tuberculosis* was used during studies (147). In the case of live virulent *M. tuberculosis*, it was shown to decrease reliance on both glycolysis and oxidative phosphorylation in infected macrophages causing them to enter a state of metabolic quiescence, while also increasing mitochondrial dependency on fatty acids for survival in infected macrophages, while heat killed *M. tuberculosis* and BCG led to increases in glycolysis (147). The reduced glycolytic rate in live *M. tuberculosis*-infected macrophages indicates the mycobacterium is subverting appropriate innate immune responses. Collectively, glycolysis, and fatty acid metabolism could be potential targets for metabolic restoration during *M. tuberculosis* infection which would promote a pro-inflammatory phenotype in infected macrophages. *B. pertussis* can also promote dysregulated glucose metabolism in the host (150). A murine model of *B. pertussis* infection demonstrated insulin resistance as the bacterium negatively regulated blood glucose homeostasis (150, 151). Host immune cells depend on circulating blood glucose for their metabolic requirements, which are particularly important in responding to infection to ensure swift activation and expansion (150). The hypoglycemic state induced during *B. pertussis* infection deprives the immune system of energy needed to create an effective immune response against the bacterium.

#### Effects of Metabolites on Immunometabolism

Cellular metabolites which may be induced by pulmonary pathogens during infection have the potential to regulate immune cell activity. LPS-stimulated macrophages were shown to increase their production of itaconate, a cellular metabolite created by diverting aconitate from the TCA cycle during inflammatory activation of macrophages (152, 153). Itaconate has been shown to have anti-inflammatory effects on macrophages by activating the anti-inflammatory transcription factor Nrf2, and acting as a succinate dehydrogenase (SDH) inhibitor leading to reduced reactive oxygen species (ROS) production and reduced IL-12, IL-1β, and IL-6 levels (153, 154). Given that gram-negative pulmonary bacteria express high levels of LPS the induction of itaconate could be a novel method used by these bacteria during chronic infection to reduce inflammatory responses of macrophages. However, itaconate itself has been shown to have antimicrobial effects by inhibiting bacterial isocitrate lyases (ICLs), enzymes involved in bacterial metabolism of fatty acids, needed for intracellular survival for many bacteria (155). *Yersinia pestis* and *Pseudomonas aeruginosa* carry genes encoding enzymes which degrade itaconate (156). This indicates that there is a complex relationship between itaconate and bacteria, and this metabolite may play different roles during acute and chronic infection. Significant work is still needed to dissect the pathways involved in itaconate's impact during pulmonary infection.

Considering the metabolic pathways that pathogens are targeting to promote immunosuppression in the host lung could potentially reveal novel targets which would improve our ability to understand and treat a wide range of infections.

It is evident the targeting of key metabolic pathways, the expansion of regulatory immune cell populations and enhanced production of associated cytokines is an advantageous method of promoting bacterial survival within the lung. Many bacterial species which invade the lung possess a multitude of methods to skew local host immune responses toward an immunosuppressive state, enabling unperturbed survival within this tissue.

## Targeting Immunosuppressive Manipulation: a Potential Therapeutic Option to Treat Pulmonary Infections

An improved understanding of the strategies employed by bacteria to subvert immunosuppression in the lung could open up new avenues for much needed therapeutic development. The development of HDTs represents an attractive approach that could be used as adjunct therapies to complement current antibiotics (157). By targeting the immunosuppressive responses hijacked by many pulmonary bacteria, key evasion methods are removed, allowing a more appropriate inflammatory immune response to ensue and effectively clear the infection.

### Small Molecule Inhibitors-Targeting Anti-Inflammatory Responses

#### IL-10 Signaling

Targeting the signaling pathways of immunosuppressive cytokines using small molecule inhibitors is an effective way to dampen down their excessive inhibitory effects. In a murine model of chronic *M. tuberculosis* infection, targeting the IL-10-STAT3 signaling pathway using an aerosolized peptide inhibitor of STAT3 led to enhanced nitric oxide synthase (NOS) and NADPH oxidase activity in conjunction with reduced arginase activity in lung homogenates, resulting in improved bacterial clearance (158). In previous studies it was shown that STAT3 signaling not only increases IL-10 production but also represses NOS in human macrophages during *M. tuberculosis* infection leading to impaired T cell responses (159, 160). This study demonstrated that even without the use of antibiotics the bacterial load in the lungs could be significantly reduced using small molecule inhibitors directed against the host immune response. Similarly, the use of a selective small molecule inhibitor of IL-10Ra in a chronic *M. tuberculosis* model led to increases in lysozyme activity in the lung resulting in reduced CFUs (158). The enhanced pro-inflammatory response seen upon IL-10 blockade is likely to be partially caused by the return of normal phagosomal maturation in innate cells such as AMs. Preventing phagosomal maturation within AMs that had engulfed *M. tuberculosis* was central to bacterial intracellular survival and IL-10 blocking allowed normal maturation to occur leading to bacterial degradation and enhanced clearance of the mycobacterium (96).

Bruton's tyrosine kinase (BTK) inhibitors which are approved for treatment of B cell lymphomas have the capacity to block both BCR signaling and STAT3 activation and are capable of reducing IL-10 production and PD-L1 expression in B cells (161). The use of these inhibitors to specifically target Breg cells could potentially be used to treat infections such as *M. tuberculosis* which has been shown to subvert Breg responses (85, 89).

#### TGF-β Signaling

Targeting TGF-β signaling using small molecule inhibitors also holds promise for improving outcomes during pulmonary invasion. *S. pneumoniae* colonization of the nasopharynx is a major pre-requisite for invasive infection and is associated with high TGF-β levels. The administration of a small molecule inhibitor of TGF-β to mice during *S. pneumoniae* colonization enhanced neutrophil influx into the nasopharynx aiding in a profound reduction in the levels of bacterial carriage compared to control mice (81).

The regulatory cytokines IL-10 and TGF-β are both required for Treg maintenance and function (162, 163), consequently targeting these cytokines can result in the depletion of Treg cells. During *M. tuberculosis* infection inhibition of TGF-β signaling using a small molecule inhibitor which targets the TGFβ type I receptor kinase, ALK5, prevented Smad3 activation leading to reduced Treg cells and enhanced Th1 responses which promoted bacterial clearance (164). These studies demonstrate the potential for transient regulation of Tregs and their associated cytokines in improving mycobacterial clearance. In *in vitro* studies, a synthetic small peptide inhibitor of TGF-β, P17, was shown to inhibit the suppressive activities of Treg cells on effector T cells (165), indicating the potential for P17 to be used to enhance protective effector T cell responses *in vivo* during infection. Interestingly P17 was developed using a phage-displayed random peptide library. It is a useful method for high-throughput screening of protein interactions and helps in identifying bioactive peptides with high affinity ligand-binding (166, 167), a similar approach may lead to the development of small peptide inhibitors against other immunosuppressive cytokines.

### Monoclonal Antibodies

Monoclonal antibodies have been in use for over four decades to treat a wide variety of human pathologies, and have revolutionized anti-cancer therapeutics (168), where these antibodies are used to target the anti-inflammatory arm of the host immune system in an attempt to promote anti-tumor immune responses. The use of monoclonal antibodies has also been investigated as method to treat infectious disease.

#### Blocking Immunosuppressive Cytokine Activities

In an experimental model of chronic tuberculosis infection, a monoclonal antibody targeting IL-10R was administered 90 days into the infection, as this time point was before loss of control of infection and when IL-10 levels peaked in this model, and weekly thereafter. Administration of the anti-IL-10Ra antibody led to increased numbers of CD4^+^ and CD8^+^ T cells infiltrating the lung and enhanced production of IFN-γ which was associated with a large reduction in bacterial burden (169). Similarly targeting TGF-β during chronic *M. tuberculosis* infection may improve effector T cell responses. *In vitro* studies using PBMCs from tuberculosis patients treated with an anti-TGF-β antibody led to reduced bacterial growth and increased IFN-γ production by these cells (170, 171). There may be a potential treatment benefit from blocking the biological functions of IL-27 in the lung post-sepsis. Cao et al. found that IL-27 regulated the increased susceptibility to secondary *P. aeruginosa* pneumonia in septic mice and promoted bacterial survival (138). During sepsis patients mount a massive inflammatory response initially. However, most patients survive the initial hyper-inflammatory period and enter into an immunosuppressed state where the individual is likely to contract and succumb to a secondary pulmonary infection (172). The inhibition of IL-27 alongside antibiotic treatment may improve survival rates. In a murine model of post-influenza secondary pneumococcal pneumonia the neutralization of IL-27 using an anti-IL-27 antibody was protective. The absence of IL-27 led to a restoration of IL-17 production from protective γδ T cells which were critical for orchestrating bacterial clearance (173).

#### Blocking Immunosuppressive Cells

Monoclonal antibodies have of course been used with much success to deplete specific immunosuppressive cell populations. One of the biggest success stories of monoclonal therapy is the development of immune checkpoint inhibitors for cancer immunotherapy. These antibodies target key immune regulating surface molecules on cells such as Tregs and MDSCs to impede their anti-inflammatory effects allowing for enhanced pro-inflammatory responses (174). Checkpoint inhibitor therapies in the context of treatment for pulmonary bacterial infections will be discussed in more detail below.

### Checkpoint Inhibitors—Targeting Immunosuppressive Cells

Checkpoint inhibitors (CPIs) which restore pro-inflammatory T cell function have been shown to prolong survival in cancer patients with various malignancies (175, 176). CTLA-4 is constitutively expressed on Treg cells and was the first immune checkpoint protein shown to inhibit T cell proliferation (177). Following on from this other immune checkpoint mechanisms such as PDL1-PD1 interactions were identified as potential targets (176). As of 2018 six immune checkpoint inhibitors have been approved for the treatment of advanced and metastatic cancers such as advanced gastric adenocarcinoma and metastatic melanoma (178).

During an infection pathogens wish to evade detection just like cancer cells and evidence suggests that these immune checkpoint pathways may also play an important role during infection to promote regulatory immune responses (175). During active tuberculosis infection there is an increase in both CTLA-4 and PD-1 on the surface of T cells and an increase in PDL-1 expression on APCs from these patients, leading to the reduced activation of an appropriate adaptive response (179). It was shown *in vitro* that blocking PD-1 using a checkpoint inhibitor led to improved *M. tuberculosis*-specific IFN-γ^+^ T cell responses (180). The blocking restored effector T cell function and reduced rates of apoptosis in these T cells (179, 180). Conflicting results were seen in PD-1 knockout mice, which had reduced survival when infected with *M. tuberculosis* (181), suggesting a possible protective role. However, it is likely complete ablation of PD-1 from the system results in an excessive inflammatory response which limits survival. During the course of *M. tuberculosis* infection there is an upregulation of PD-1. It is possible that long-term disease results in its overexpression causing the dampening of protective inflammatory immune responses (179). The short term inhibition of PD-1 during infection could improve pro-inflammatory responses while reducing the likelihood of hyper-inflammation seen in the PD-1 knockout mice. Clinical trials have been carried out using CPIs in the treatment of viral infections such as Human immunodeficiency virus (HIV) infection (182). A phase II clinical trial using anti-PD-L1 alongside anti-retroviral therapy (ART) in HIV patients was stopped early due to retinal toxicity observed in a simultaneous macaque study (183). However, two of the six patients involved in the trial showed increased antigen-specific CD4^+^ and CD8^+^ T cells indicating the potential for CPIs to enhance effector T cell responses during infection.

The blockade of checkpoint molecules can lead to immune-related adverse effects as these molecules are also involved in regulating immune tolerance to prevent immunopathology and autoimmunity (175). These monoclonal antibodies are used in severe cases of cancer, therefore concerns still remain over their use for infection. Further research is critical into the efficacy of CPIs as potential therapies for improving various pulmonary infection outcomes.

### Targeting Metabolic Changes Induced During Infection

Metabolic reprogramming is recognized as a hallmark of cancer (184, 185) and targeting metabolic pathways has been used for the treatment of various cancers to interfere with tumor progression (184, 186). During bacterial infections of the lung the metabolic pathways of many immune cells are re-programmed to a more immunosuppressive phenotype, benefitting the bacteria and leading to chronic infection. Targeting these pathways to reverse this reprogramming has the potential to promote more pro-inflammatory responses and improve bacterial clearance.

#### Targeting Fatty Acid Oxidation

In murine tumor models it has been shown that tumor infiltrating MDSCs increased their fatty acid uptake and increased fatty acid oxidation (FAO) (187). Blocking FAO using the inhibitor Etomoxir, a carnitine palmitoyltransferase-1 (CPT-1) inhibitor which prevents fatty acid transport into the mitochondria for further metabolism, reduced the immunosuppressive effects of these cells leading to reductions in Treg numbers, enhanced effector T cell proliferation, and IFN-γ production which delayed tumor growth (187). FAO inhibition could also be used to directly reduce the number of Treg cells as FAO has been shown to be central to the differentiation of Treg cells (188). FAO inhibition could be an innovative approach to reduce the anti-inflammatory effects of both MDSCs and Tregs during pulmonary infection to improve bacterial clearance and current antibiotic treatments. However, it should be noted that CD8^+^ memory T cell also use FAO as an energy source (189), the inhibition of FAO could have implications for these cells resulting in defective memory responses which could have negative impacts on infection responses.

#### Skewing Macrophage Phenotypes

The targeting of metabolic pathways in macrophages could also be potentially used as a therapeutic approach during pulmonary bacterial infection to skew macrophages toward a more protective M1 phenotype. Itaconate promotes macrophage switching from a pro- to an anti-inflammatory state and the metabolite is emerging as a crucial determinant in macrophage activity (190). The inhibition of itaconate may be of benefit in chronic bacterial lung infection where enhanced M1 responses could improve bacterial clearance. Irg1 signaling regulates itaconate production, the blocking of Irg1 may be a method of reducing itaconate to promote succinate dehydrogenase (SDH) activity enabling M1 responses against invading bacteria and impairing their ability to alter macrophage phenotypes to an M2 state. In a murine model of *M. tuberculosis* using Irg1^−/−^ mice that do not produce itaconate, the mice exhibited an increase in neutrophil influx and pro-inflammatory cytokine production compared to WT mice (191). However, these mice succumbed due to immunopathology of excessive inflammation. It needs to be established if targeting Irg1/itaconate in a specific cell population only could prevent immunopathology.

### Targeting Immunosuppressive Responses Using RNA Interference

RNA interference is a method of gene silencing mediated through the use of small interfering RNA (siRNA) or microRNA (miRNA). Studies using siRNA demonstrate how the immune response can be modified into a more pro-inflammatory phenotype during chronic *M. tuberculosis* infection (192). In mice chronically infected with *M. tuberculosis* the intrapulmonary administration of siRNA that specifically targets TGF-β leads to reduced bacterial load in the lungs and increased production of pro-inflammatory cytokines such as TNF (192). The silencing of the *tgfb1* gene directly in the lungs improves the antimicrobial capacity of the host.

Another promising method of RNA interference during bacterial infection is the use of miRNAs. The silencing of certain regulatory miRNA have the potential to improve bacterial clearance by promoting pro-inflammatory immune cell responses. The inhibition of miR-328 could improve bacterial clearance during a non-typeable *H. influenzae* (NTHi) infection (193). *In vitro* studies using murine macrophages and neutrophils demonstrated miR-328 inhibition using an antagomir of miR-328 prior to NTHi infection promoted enhanced phagocytosis, increased ROS production and improved bacterial killing in both cell types. Further *in vivo* studies found boosting miR-328 downregulation by intra-tracheal administration of the miR-328 inhibitor enhanced bacterial killing rates (193). This method of miR-328 inhibition could potentially be used during chronic pulmonary infection to remove bacterial-induced immunosuppression in innate cells such as macrophages. The use of RNA interference in the treatment of infection is an area of ongoing research.

### Repurposing Chemotherapeutic Drugs to Target Immunosuppressive Responses

The repurposing of chemotherapeutic drugs developed for other diseases is an avenue of research being investigated to target regulatory immune cells induced locally during lung infection.

#### Targeting MDSCs

Chemotherapeutic drugs that target MDSCs could potentially be used in combination with standard antibiotic treatments to improve the immune response against chronic pulmonary bacterial infections. All-trans retinoic acid (ATRA) is an approved anti-cancer treatment, which leads to the maturation-induced ablation of MDSCs that often infiltrate tumors (194, 195). Ablation of MDSCs in a murine model of tuberculosis was carried out using ATRA treatment (196). Mice that were administered ATRA after tuberculosis infection had reduced numbers of MDSCs in their lungs, lower bacterial loads and improved effector T cell numbers (196). Sorafenib is an immunotherapeutic anti-tumor drug used against hepatocellular carcinoma (197). Sorafenib downregulates the MDSC population to promote a more pro-inflammatory anti-tumor environment (197). Drugs such as Sorafenib could potentially be used to reduce MDSCs during pulmonary infections.

#### Targeting Tregs

Elevated Treg levels have been associated with poor prognosis in certain types of cancer (177). Many anti-cancer therapies, such as checkpoint inhibitors mentioned earlier target Treg cells to improve outcomes in advanced cancers (178). A common chemotherapeutic used against glioblastoma is temozolimide (TMZ). In a rat glioma model, low doses of TMZ selectively inhibited the activity of Treg cells (198). In humans with advanced chemotherapy resistant cancers low iterative oral doses of cyclophosphamide resulted in a selective reduction of circulating Treg cells (199) while other T cell subset numbers and functions were preserved. It would be interesting to determine if low doses of TMZ administered to the airways during pulmonary infection could have a similar effect, selectively depleting Tregs thus promoting more pro-inflammatory responses and potentially improving rates of bacterial clearance.

The anti-cancer drug denileukin diftitox (DD) is used to treat cutaneous T cell lymphomas (200, 201). The drug is a synthetic protein that combines IL-2 and Diphtheria toxin, which acts by binding the IL-2R and delivering the diphtheria toxin to the cell resulting in cell death (201). In a murine model of tuberculosis, DD treatment during infection depleted both Treg cells and MDSCs from the lung of infected mice leading to improved bacterial clearance compared to an untreated group (202). Further investigation into the use of DD in the treatment of other chronic pulmonary bacterial infections is needed to test its efficacy. However, DD treatment is associated with adverse effects such as capillary leak syndrome, hypoalbuminemia, and visual changes which have obvious implications for the use of this treatment for infection.

The repurposing of cancer chemotherapeutics is advantageous as many of these drugs have already gone through testing, have known pharmacological properties and have reduced costs and development times compared with the production of novel antimicrobial drugs. However, because the use of chemotherapeutics to deplete immunosuppressive cell populations have the risk of promoting excessive inflammation and autoimmunity, these treatments should be approached with great caution and intensive safety testing is needed to investigate the off-target effects they may have during infection.

**Figure 2:**
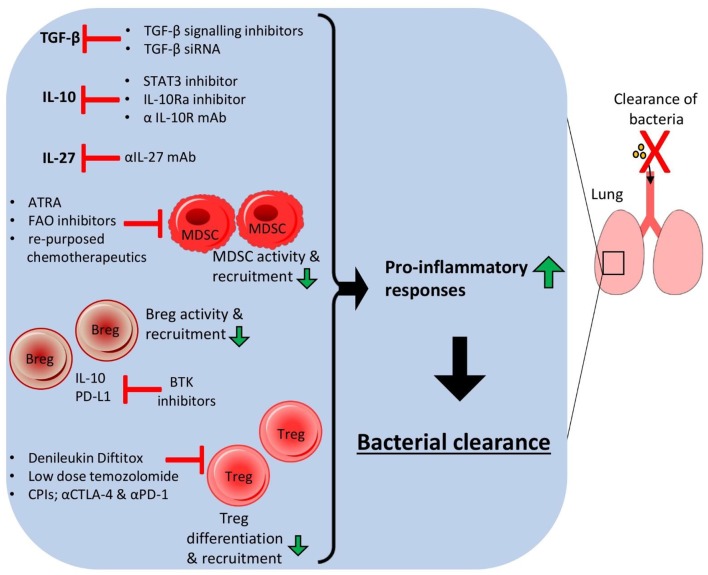
Host-directed therapies targeting immunosuppressive responses in the lung lead to bacterial clearance. Host-directed therapies (HDTs) that target anti-inflammatory immune responses in the host lung can improve bacterial clearance and reduce chronic infection. Small molecule inhibitors of cytokine signaling pathways, monoclonal antibodies or siRNA could be used to reduce the levels of anti-inflammatory cytokines such as IL-10, TGF-β and IL-27, allowing higher pro-inflammatory cytokine production and reduced activation and expansion of anti-inflammatory cell populations. HDTs targeting specific anti-inflammatory cells could also improve infection outcome by depleting these cell populations.

The ability of many lung-associated bacterial pathogens to induce immunosuppression to promote their survival can potentially be ablated by a number of HDTs targeting various aspects of the local regulatory host immune response such as regulatory cell populations, anti-inflammatory cytokines and associated metabolic signaling pathways (Figure 2). Combining various methods could potentially be used to improve pulmonary infection outcomes without relying on antibiotic use. The use of drugs targeting key metabolic pathways such as FAO and cellular interactions such as checkpoint molecules could drastically reduce the ability of bacterial pathogens to promote local immunosuppressive responses thus boosting protective inflammatory responses and associated bacterial elimination. The repurposing of drugs such as anti-cancer chemotherapeutics could therefore open up novel treatment options for bacterial clearance from the lung and improve MDR infections.

## Targeting Immunosuppressive Responses to Improve Vaccine Efficacy

HDTs that specifically target the immunosuppressive arm of the pulmonary immune system during bacterial infection have the potential to undo the detrimental anti-inflammatory responses induced by bacteria, and “re-inform” the local immune response to react in a more inflammatory manner. It is possible that similar strategies could be implemented to potentially improve vaccine function by altering the immune microenvironment in which the vaccine is administered.

The BCG vaccine is currently the only available prophylactic vaccine against *M. tuberculosis*. In response to BCG vaccination there is an expansion of Treg cells and an increase in the production of IL-10 alongside the expansion of Th1 responses (203). This is likely contributing to the lack of efficacy of the BCG vaccine against pulmonary TB. In vaccination studies using IL-10 KO mice it was demonstrated that KO mice vaccinated with the BCG vaccine had enhanced DC activation and Th1 responses compared to vaccinated wild-type mice (204). DCs isolated from vaccinated IL-10 KO mice co-cultured with CD4^+^ T cells induced significantly higher levels of IFN-γ production compared to when DCs from vaccinated WT mice were used, indicating IL-10 was suppressing these protective inflammatory responses (204). This demonstrated that IL-10 signaling was contributing to the DC dysfunction in BCG vaccination impairing these cells abilities to promote inflammatory T cell responses. Other studies showed that mice treated with IL-10R neutralizing antibodies during BCG vaccination were better protected against subsequent *M. tuberculosis* infection compared to mice who were not pre-treated with the antibody. This was due to enhanced antigen-specific IFN-γ and IL-17A responses (205, 206). These studies illustrate the potential for controlled short-term regulation of IL-10 during vaccination to establish better T cell-mediated immunity and improve vaccine efficacy.

Wu et al. demonstrated that the BCG strain of mycobacterium used in the vaccine can actually induce transcription of the immunoregulatory microRNA miR-21 in murine lungs, which may also contribute to vaccine inefficacy (207). miR-21 led to reduced IL-12 expression in macrophages, enhanced DC apoptosis, and suppressed Th1 responses. The temporal and specific blocking of miR-21 during immunization therefore could potentially improve anti-*M. tuberculosis* vaccines. There is now a growing interest in using anti-miR compounds to improve disease outcomes (208). In anti-cancer vaccine studies the use of small molecule PI3K inhibitors during vaccination was shown to improve vaccine efficacy and promote pro-inflammatory Th1-skewed responses by preventing IL-10 production (209). In a murine cancer model mice with solid tumors were given a DC vaccine consisting of DCs that had been pre-treated with a PI3K inhibitor, TLR5 agonist, and tumor antigen. The PI3K inhibitor prevented anti-inflammatory signaling from TLR activation which reduced IL-10 production and enhanced IL-12 production from the DCs. PI3K inhibition heightened the antitumor properties of the DC vaccine by relieving suppressive signals that restrict DCs abilities to induce potent antitumor T-cell responses.

The administration of an anti-TGF-βR1 signaling inhibitor, D4476, 24 h post-vaccination with BCG improved protective Th1 immune responses against subsequent pulmonary *M. tuberculosis* infection (210). This inhibitor was given in conjunction with a Th2 cell inhibitor. Vaccinated mice that received the immunomodulatory inhibitors had reduced bacterial load and better IFN-γ^+^ T memory cell responses upon infection compared to mice that did not receive the inhibitors (210).

These studies indicate the inhibition of the anti-inflammatory arm of the immune response during vaccination may have the potential to improve vaccine efficacy. This method of inhibition could be used to improve current vaccines against pulmonary bacteria that exploit the anti-inflammatory immune response during infection, ensuring more pro-inflammatory responses upon exposure. Further research is needed into methods of suppressing particular cells and cytokines of the regulatory pulmonary immune response to improve vaccine outcomes against infectious disease.

## Conclusion and Future Perspectives

The increasing incidence of multidrug resistance in pathogenic bacteria and the slow pace of novel antibiotic development threatens our ability to treat these bacterial infections effectively. Bacterial lung infections are a major concern as any damage to the lung dramatically impacts the overall health and survival of a patient. Host-directed therapeutic strategies targeting bacterial-induced anti-inflammatory immune responses must be considered as viable adjuncts to standard antimicrobial treatment. Additionally, there is the potential for targeting immunosuppression to improve vaccine efficacy for current and next-generation vaccines targeting bacterial species. However, there are challenges in bringing these new approaches to the clinic. A better understanding of the host-bacterial interaction during pulmonary infection is needed, in order to identify how these immunoregulatory responses are being induced and the impacts dampening these responses could have.

Any therapeutic intervention that promotes the pro-inflammatory immune response has a high risk of inducing excessive inflammation which has the potential to lead to cytokine storm and be detrimental to the host (157). The promotion and recruitment of enhanced pro-inflammatory cell populations, such as neutrophils, have been linked to increased incidence of tissue injury (211). The production of reactive oxygen species (ROS) by phagocytic cells plays a fundamental role in the removal of pathogens from the host. However, the dampening of regulatory responses through the use of HDTs discussed here have the potential to result in excessive and uncontrolled oxidative stress and ROS production at the site of infection (211). Excessive production of ROS has been linked to reduced CD8^+^ T cell function during viral infection (212), and impaired immune responses in the lungs of cystic fibrosis patients where the free radicals generated during chronic inflammation cause oxidative damage of pulmonary proteins, likely contributing to the decline of lung function in CF patients (213). Unrestricted oxidative stress has been associated with infection complications and the induction of diseases such as neurodegenerative and cardiovascular disorders, and cancer (214). Serious consideration of potential immunopathological side effects must be taken into account when considering these HDTs as possible treatment options for MDR lung infections.

The inhibition of the anti-inflammatory response must be temporal and precise to prevent these hyper-inflammatory responses and off-target effects. By gaining a greater understanding of the immunoregulatory mechanisms being hijacked by pulmonary bacteria we may be able to better tailor antibacterial therapies to avoid off-target consequences. The optimal timing of when to administer these HDTs needs to be elucidated, meaning we need further investigation into how and when pulmonary bacteria are inducing these anti-inflammatory responses in order to target them at the correct point of infection. Improving delivery of anti-inflammatory inhibitors may reduce their off-target effects and could limit inhibition to certain target cells. The use of β-glucan nanoparticles, for example could be used to specifically target macrophages (215). These nanoparticles can encapsulate small molecule inhibitors or siRNA to improve their delivery into cells. The β-glucan outer shell promotes receptor-mediated uptake by phagocytic cells that express β-glucan receptors (215). This approach could be used to specifically deliver HDTs into macrophages in the lung to inhibit early immunosuppressive responses.

The study and development of novel HDTs against pathogen-induced anti-inflammatory immune responses during pulmonary infection represents a method of strategically modifying the immune response to improve current treatments and vaccine efficacy against many multidrug-resistant bacteria. In this era of re-emerging infectious diseases as a consequence of increases in antimicrobial resistance, the development of alternative antimicrobial strategies is imperative to tackle this major healthcare challenge.

## Author Contributions

AK and RM conceived the idea for the manuscript. AK drafted the manuscript. RM edited and added the valuable insights into the manuscript.

## Conflict of Interest

The authors declare that the research was conducted in the absence of any commercial or financial relationships that could be construed as a potential conflict of interest.
